# Kienböck’s Disease Following a Hypersupination Injury: A Case Report

**DOI:** 10.7759/cureus.59467

**Published:** 2024-05-01

**Authors:** Anmol Singh, Ryan Reese

**Affiliations:** 1 Family Medicine, University of Maryland Medical Center, Baltimore, USA; 2 Sports Medicine, University of Maryland Medical Center, Baltimore, USA

**Keywords:** kienböck’s disease, muskuloskeletal mri, hand casting, sports medicine, avascular necrosis of lunate, trauma, hypersupination, chronic wrist pain, avascular osteonecrosis

## Abstract

Wrist pain is a common presentation in primary care clinics. Chronic pain after trauma with non-acute radiographs requires careful physical examination and a case-specific workup. We present a case of a 32-year-old female evaluated at the primary care clinic with two months of left wrist pain after a hypersupination injury that was found to be secondary to avascular necrosis of the lunate on the left wrist with no radiographic signs of fracture or focal sclerosis on plain films. This case demonstrates the importance of identifying less common chronic wrist pain etiologies.

## Introduction

Chronic wrist pain limits patients’ activities of daily living and requires careful diagnostic evaluation [1]. More common presenting pathologies include scaphoid fracture nonunion, thumb carpometacarpal joint osteoarthritis, scapholunate instability, triangular fibrocartilage complex injury, De Quervain tenosynovitis, extensor carpi ulnaris tendinopathy, carpal tunnel syndrome, and ganglion cysts. Overuse injuries may be associated with tendinopathies without osseous involvement. The first-line investigation depends on the clinical exam, and patients may be referred for additional imaging beyond radiographs, such as ultrasound scans, nerve conduction studies, electromyography, or magnetic resonance imaging (MRI), depending on the clinical picture [1]. However, the patient’s history may trigger warning signs of rarer etiologies of wrist pain [2]. We report a case of osteonecrosis of the lunate (Kienböck’s disease) that was not noted on initial radiographs.

## Case presentation

A 32-year-old right-handed female with a medical history of polycystic ovarian syndrome presented to the primary care sports medicine clinic with left wrist pain for two months after hypersupinating her wrist to prevent her young daughter from falling. She felt pain over the dorsal aspect of her wrist with wrist extension. The injury happened in India, where she had radiographs performed three weeks after the initial injury. She was told abroad that she had an unspecified hairline fracture and was in a cast for six weeks. Her pain persisted after the casting, and she used an over-the-counter thumb spica splint for comfort before presentation. She works as a neonatal intensive care unit (NICU) nurse, and her pain limited her ability to provide patient care.

She denied erythema, rashes, fevers or chills, cardiorespiratory complaints, weight loss, weight gain, and myalgias/joint pain outside of left wrist pain. The right wrist was unaffected. She had been able to perform her activities of daily living due to her right-hand dominance. Her vital signs were within normal limits (Table 1). On physical examination, she was well-appearing and alert. Her left wrist exam was pertinent for dorsal swelling with no ecchymosis, erythema, or rashes. She was tender to the radiocarpal joint with no anatomic snuff box tenderness or tenderness to the first dorsal compartment or metacarpal bones. Her active flexion and extension were reduced by 30 degrees each compared to the normal range of motion (Table 2). Her left wrist strength was 5/5 in flexion, extension, supination, and pronation. Grip strength was 5/5. Special wrist tests revealed negative Watson's, Finkelstein’s, and triangular fibrocartilage complex (TFCC) grind tests.

**Table 1:** The patient's vital signs

Vital signs	Values	Reference range
Blood pressure	129/79	<140/90
Pulse	92 bpm	60-100 bpm
Temperature	36.8 °C	36-37.2°C
Weight	195 lbs	125.3-169.3 lbs
Height	1.753m	n/a
BMI	28 kg/m²	18.5 to 24.9 kg/m²

**Table 2:** The patient's range of motion exam

	Range of motion	Normal range of motion
Active flexion	30°	80°-90°
Active extension	30°	70°-90°
Active pronation	90°	90°
Active supination	90°	90°

**Figure 1:**
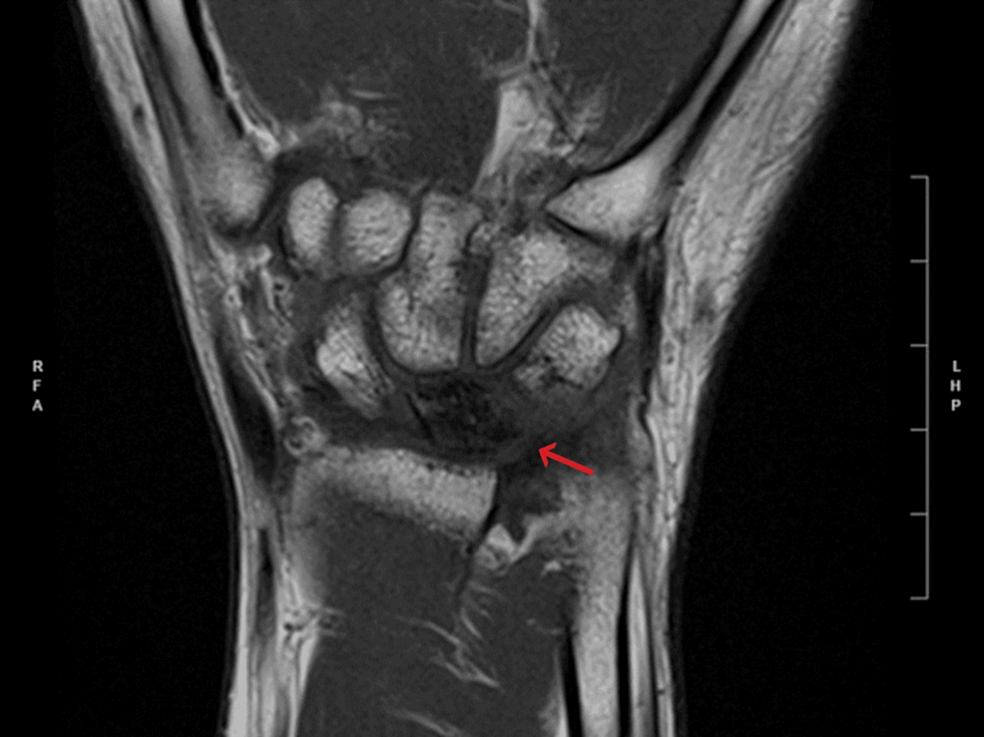
T1 coronal MRI The arrow corresponds to a diffuse low signal in the lunate bone. Image #10/19; spin echo (SE): 10001

**Figure 2:**
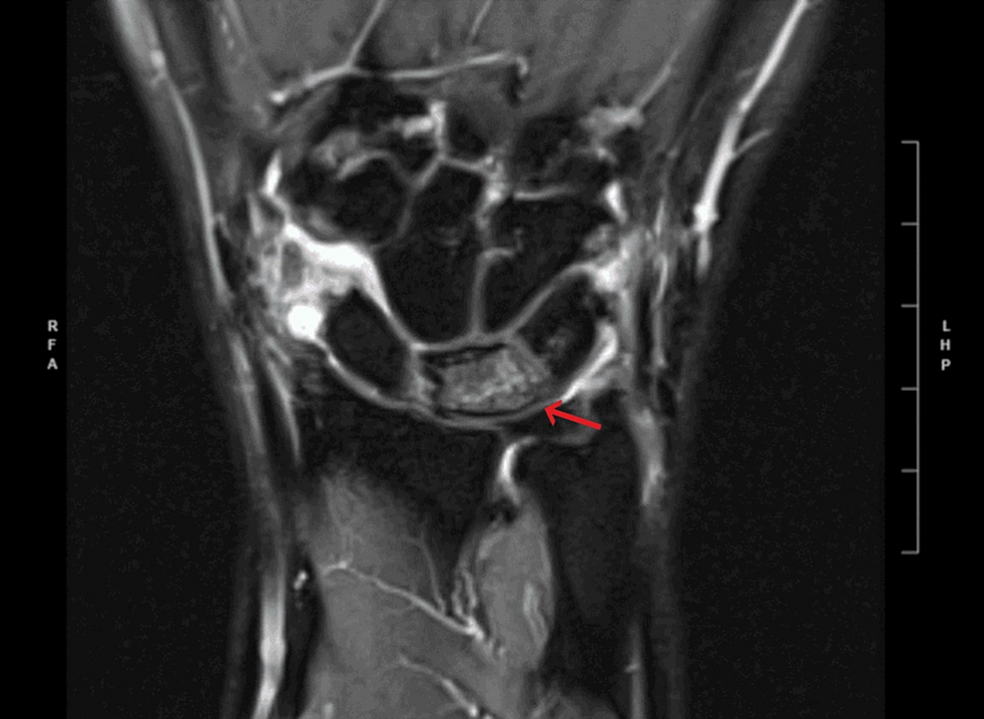
T2 Coronal MRI The arrow corresponds to diffuse increased signal intensity in the lunate bone compared to the T1 series, which may be representative of avascular necrosis. Image #11/19; spin echo (SE): 6001

**Figure 3:**
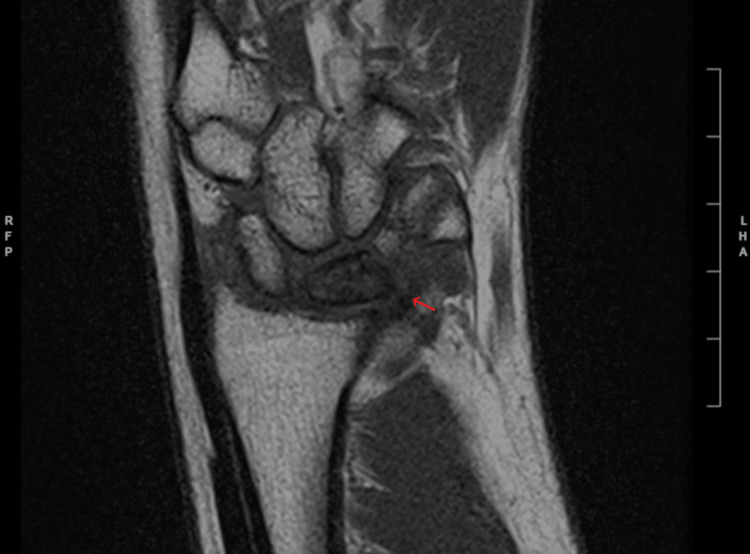
Post-treatment T1 coronal MRI The arrow corresponds to the diffuse low signal intensity of the lunate, which is similar to the previous MRI. Image # 10/19; spin echo (SE): 3001

**Figure 4:**
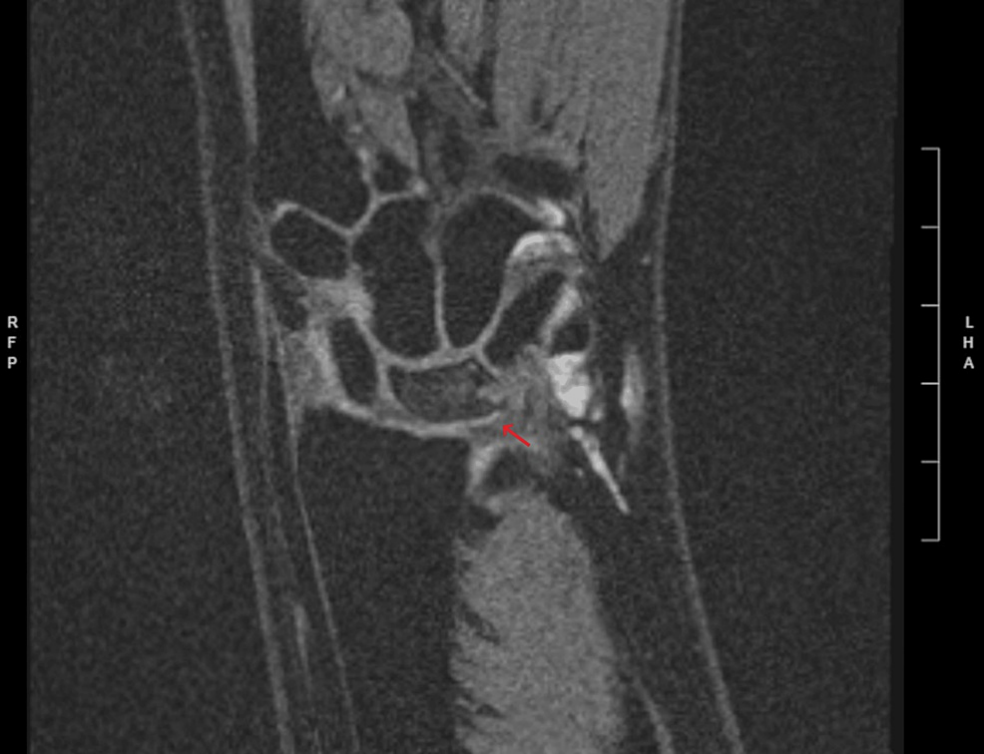
Post-treatment T2 coronal MRI The arrow corresponds to diffuse increased signal intensity, which is similar to prior T2 coronal MRI. Image #10/19; spin echo (SE): 4001

Her radiographs from India, dated October 21, 2023, were brought for review, but the images were not scanned into the patient chart. There was no evidence of fracture, dislocation, or malalignment. She was provided with a cock-up wrist splint during the initial clinic visit. An MRI of the wrist without contrast was ordered, which revealed a diffuse low signal throughout the lunate with an increased T2 signal on fluid-sensitive sequences, suggesting avascular necrosis of the lunate (Figures 1-2). There was also non-specific carpal joint effusion posteriorly, which may reflect sequelae of trauma or acute injury. These findings suggested her chronic left wrist pain was likely due to avascular necrosis of the lunate, also known as Kienböck’s disease. The patient responded well to a follow-up short arm cast under the hand surgeon's supervision, which was transitioned to a brace, eventual occupational therapy, and a targeted home exercise plan with resolution of pain. Follow-up MRI imaging after casting re-demonstrated avascular necrosis, but the patient had symptomatic relief and no further imaging was obtained (Figures 3-4).

## Discussion

Osteonecrosis of the lunate, which is described as a breakdown of the lunate, is associated with fragmentation and collapse of the lunate [3]. The etiology is unknown and is multifactorial, with possible predisposing factors including vascular perfusion alterations, microtrauma, variable lunate anatomy, and altered loading. Lunate ischemia related to one or more of these factors may lead to vascular disruption that contributes to osteonecrosis [4]. Patients with Kienböck’s disease typically present with unilateral wrist pain in their dominant hand with an insidious onset and no history of major trauma. They tend to have difficulty with hand grip and a decreased range of motion [5]. It is possible, as in the case outlined, for there to be cases where a predisposing event leads to microtrauma that is associated with the development of the disease state. It also primarily affects young to middle-aged adults.

Diagnosis starts with clinical assessment and radiography, which may reflect sclerosis and flattening of the lunate, and the anatomy is further characterized by MRI to evaluate signal changes and sclerosis with assessments of lunate collapse, carpal height, joint spaces, and swelling [6]. This allows for the extent of avascular necrosis to be evaluated and to evaluate adjacent soft tissue structures. The Lichtmann classification is utilized for staging patients with Kienböck’s disease and utilizes wrist radiographs as part of the classification [7]. In stage I disease, signs of avascular necrosis of the lunate are only seen on MRI. In stage II, there are radiographic signs of lunate sclerosis (Table 3). In stage IIIa, there is lunate collapse without scaphoid rotation, whereas in stage IIIb, there is lunate collapse with fixed scaphoid rotation. In stage IV disease, there is lunate collapse with fixed scaphoid rotation and degeneration of adjacent intercarpal joints (Table 3).

**Table 3:** Lichtmann stages of classification Classification depicting progressive stages of lunate bone osteonecrosis [7]

Lichtmann stage	Radiographic findings
I	Changes only noted on MRI
II	Lunate sclerosis
IIIa	Lunate collapse without scaphoid rotation
IIIb	Lunate collapse with fixed scaphoid rotation
IV	Lunate collapse with scaphoid rotation and degeneration/osteoarthritis of intercarpal joints adjacently

Capo et al. outlined one case in which a 55-year-old woman sustained a distal radius fracture treated with closed reduction and splinting, which was appropriately treated and healed on radiography, but the patient developed ulnar pain four months after the injury. Radiography taken at the six-month mark demonstrated lunate fragmentation, and a follow-up MRI confirmed Kienböck’s Disease. Our case shares similarities in that the patient developed osteonecrosis as a sequala of low-energy microtrauma. The report by Capo et al. discussed how the alignment of the carpus in some patients may also predispose the lunate to uneven bone stress. Anatomic variation among patients may put certain patients at risk of developing this disease. In that case, similar to ours, the patient pursued non-surgical management, including rest and observation, with the eventual improvement of pain symptoms.

Treatment options depend on disease severity, the clinical course, and the patient’s age and medical history [8]. Options range from conservative management with immobilization and anti-inflammatory therapy to orthopedic surgery, which can include revascularization, joint leveling/lunate unloading, radial shortening osteotomy, capitate shortening, and wrist salvage. Less severe cases, such as the one described, tend to favor conservative therapy such as immobilization, with the potential for lunate revascularization or other outlined treatments as future options. Activity modification and anti-inflammatory medications can also be utilized to preserve function [8]. 

The prognosis of lunate osteonecrosis depends on the staging, which evaluates the extent of osteonecrosis and joint damage. Although, early diagnosis and intervention can help prevent disease progression, complex and prolonged cases may lead to chronic pain and joint stiffness [5]. 

Omar et al. outlined one case in which a 17-year-old male presented with atraumatic left wrist pain with radiographic findings demonstrating densification of the lunate with a flattened appearance of the scaphoid with fixed rotation and no arthritic changes [2]. An MRI confirmed signs of Kienböck's disease, including fragmentation of the lunate and T2 signal hyperintensity. In this case, the patient, along with his family, declined surgery, which was offered, and elected for splinting. The follow-up was not outlined in the case, but this patient outlines an example of Lichtmann stage IIIb disease in which surgery may be indicated. In opposition to this, our case is one in which initial radiographs did not demonstrate radiographic findings of osteonecrosis, such as lunate sclerosis, and conservative therapy was recommended.

Another advantage of MRI is in cases where the differential diagnosis is unclear after a radiograph. Lunate bone density may be transiently increased as a result of microtrauma but without clinical osteonecrosis [8]. Fragmentation of the lunate may also be noted in nonunion after a traumatic fracture. Getting an MRI allows for a more specific diagnosis when the potential etiology is broad. 

Another mimic is scapholunate ligament instability [1]. This ligament is intrinsic, meaning it directly connects the two carpal bones. It typically tears during a fall, and patients present with tenderness to palpation dorsally over the scapholunate ligament interval. Patients may have a positive Watson shift test in which an examiner would grasp the patient's wrist over the scaphoid tubercle in slight extension and move the wrist from ulnar to radial deviation. The test is positive if a clunk is felt and the patient experiences pain [1]. Radiography for diagnosis should include three views of the bilateral wrist plus clenched fist views.

## Conclusions

Family medicine physicians routinely evaluate and treat patients with unilateral wrist pain related to acute injury or chronic trauma. Identifying avascular necrosis as a rare but potential diagnosis may shorten the timeline for specialty care and treatment. Family physicians need to be able to determine whether a fracture-negative wrist pain presentation necessitates further imaging or referral based on history and a physical exam. Early-stage Kienböck’s disease is not appreciable on conventional radiography, and exam findings such as weakened grip strength, restrictions in range of motion, and carpal bone tenderness or effusions may suggest further imaging with an MRI to evaluate for vascular abnormalities. Chronic untreated Kienböck’s disease may lead to the progression of the disease and the expansion of osteonecrosis, with eventual bony collapse and sclerosis. Providing a comprehensive examination for patients with wrist pain may identify those at risk for rare, more urgent musculoskeletal pathologies and improve their recovery potential. 

## References

[1] Hemmati S, Ponich B, Lafreniere AS, Genereux O, Rankin B, Elzinga K (2024). Approach to chronic wrist pain in adults: review of common pathologies for primary care practitioners. Can Fam Physician.

[2] Omor Y, Nassar I, Ajana A, Moatassimbillah N (2015). Kienböck's disease: a case report. Pan Afr Med J.

[3] Capo JT, Shamian B, Preston J (2013). Osteonecrosis of the lunate following low-energy trauma: a case report. JBJS Case Connect.

[4] Nakanishi A, Yajima H, Kisanuki O (2014). Post-traumatic osteonecrosis of the lunate after fracture of the distal radius. J Plast Surg Hand Surg.

[5] Rioux-Forker D, Shin AY (2020). Osteonecrosis of the lunate: Kienböck disease. J Am Acad Orthop Surg.

[6] Camus EJ, Van Overstraeten L (2022). Kienböck's disease in 2021. Orthop Traumatol Surg Res.

[7] Lichtman DM, Pientka WF 2nd, Bain GI (2017). Kienböck disease: a new algorithm for the 21st century. J Wrist Surg.

[8] Fontaine C (2015). Kienböck's disease. Chir Main.

